# CRISPR/Cas9-Mediated Zebrafish Knock-in as a Novel Strategy to Study Midbrain-Hindbrain Boundary Development

**DOI:** 10.3389/fnana.2017.00052

**Published:** 2017-06-30

**Authors:** Gokul Kesavan, Avinash Chekuru, Anja Machate, Michael Brand

**Affiliations:** Biotechnology Center and DFG-Research Center for Regenerative Therapies Dresden, Technische Universität DresdenDresden, Germany

**Keywords:** CRISPR/Cas9, midbrain-hindbrain boundary (MHB), knock-in reporter, zebrafish and transgenesis

## Abstract

The midbrain-hindbrain boundary (MHB) acts as an organizer and controls the fate of neighboring cells to develop into either mesencephalic (midbrain) or metencephalic (hindbrain) cells by secreting signaling molecules like Wnt1 and Fgf8. The zebrafish is an excellent vertebrate model for studying MHB development due to the ease of gene manipulation and the possibility of following cellular dynamics and morphogenetic processes using live imaging. Currently, only very few reporter and/or Cre-driver lines are available to study gene expression at the MHB, hampering the understanding of MHB development, and traditional transgenic technologies using promoter/enhancer fragments or bacterial artificial chromosome (BAC)-mediated transgenesis often do not faithfully recapitulate endogenous expression patterns. In contrast, CRISPR/Cas9-mediated genome editing technology now provides a great opportunity to efficiently knock-in or knock-out genes. We have generated four CRISPR/Cas9-based knock-in fluorescent reporter lines for two crucial genes involved in MHB development, namely *otx2* and *pax2a*. The coding sequences of the reporters were knocked-in upstream of the corresponding ATG and are, thus, under the control of the endogenous promoter/enhancer elements. Interestingly, this strategy does not disturb endogenous gene expression. Using the fast maturing fluorescent protein reporter, Venus, enabled us to follow MHB development using cell tracking and live imaging. In addition, we show that these reporter lines label various neuronal and glial cell types in the adult zebrafish brain, making them highly suitable for investigating embryonic and adult midbrain, hindbrain, and MHB development.

## Introduction

The boundary between the midbrain (mesencephalon, mes) and the hindbrain (metencephalon, met) is essential for establishing a clear demarcation between the midbrain and the anterior hindbrain. This midbrain-hindbrain boundary (MHB), also known as the isthmic organizer or midbrain-hindbrain organizer, acts as a local signaling center (Wurst and Bally-Cuif, 2001; Raible and Brand, 2004; Rhinn et al., 2006; Dworkin and Jane, 2013). The MHB forms at the interface of two transcription factor domains in the neural plate epithelium, namely the anterior Otx domain and the posterior Gbx domain (Rhinn et al., 2003). This interface is established by mutual transcriptional repression and initiates formation of the prospective MHB. Successively, morphogens such as Wnt, Fgf, and transcription factors like Engrailed1/2, and Pax2/5/8 induce the formation of the MHB, and their subsequent interplay is critical for maintenance of the MHB (Rhinn and Brand, 2001; Wurst and Bally-Cuif, 2001; Raible and Brand, 2004; Rhinn et al., 2006; Dworkin and Jane, 2013). The above-mentioned factors (Otx, Gbx, Wnt, Fgf8, Pax, and Eng) form the core of the MHB signaling machinery and a disruption of any of these factors interferes with the formation and function of the MHB.

Several model organisms, including chicken, mouse, and zebrafish, have been extensively used to understand the complex network of genes and their interactions during MHB development (Martinez-Barbera et al., 2001; Raible and Brand, 2004; Zervas et al., 2004; Rhinn et al., 2006; Sunmonu et al., 2011; Tossell et al., 2011). The zebrafish model has been instrumental in the identification of several genes that are essential for MHB development due to the availability of numerous mutants obtained from large-scale mutagenesis screens (Brand et al., 1996; Schier et al., 1996).

The zebrafish is an ideal model to study vertebrate brain development, and especially patterning in central nervous system, beginning from the neural plate stages because of the following reasons. (A) Fertilization is external and a single female can produce several hundred eggs (>200) in a single spawning; (B) the transparent nature of the embryos allows real-time imaging of the developing embryos; (C) fast embryonic development—a fertilized egg develops into a larva with most organs fully formed within 3 days; and (D) the possibility of manipulating genes and the ease of performing both loss- and gain-of-function experiments. Besides neurobiology studies in the embryo and larva, the adult zebrafish brain has widespread proliferative neural stem cells (neurogenic zones) distributed along the rostro-caudal axis (Adolf et al., 2006; Grandel et al., 2006). The zebrafish has also become a valuable model system for understanding neural stem cell heterogeneity, adult neurogenesis under homeostasis and injury, and regeneration (reviewed in Kizil et al., 2012; Grandel and Brand, 2013; Alunni and Bally-Cuif, 2016). Therefore, the ability to combine genetic manipulations with the application of advanced microscopic techniques, both in developing and adult animals, makes zebrafish a unique model to study vertebrate neurogenesis.

Effective study of adult neurogenesis or regeneration often requires labeling of specific cell types such as stem cells or neurons. The midbrain tectum, with its stratified cellular organization, is not only structurally similar to the mammalian cortex but is also involved in visual input processing and co-ordination of goal directed movements. Thus, it collectively acts as a major visual processing center in the brain. To successfully understand visual processing mechanisms, it is imperative to label individual cells or a group of cells in a neuronal network (Robles et al., 2011), and a majority of the previous studies mostly relied on neuroanatomical studies in other teleosts for such insight (Meek and Schellart, 1978; Meek, 1981). Nevertheless, few studies were successful in labeling cell populations of the midbrain tectum using Tol2-based transgenesis or gene-trap based random integration methods (Scott and Baier, 2009; Muto et al., 2013). Until recently, genetic manipulations in the zebrafish mainly entailed constitutive mutants generated using random mutagenesis screens or transposon-mediated random integration of transgenic constructs. Although, cell-specific promoters of interest would still be active in the adult fish, random integration often results in ectopic expression and/or gene silencing, thereby limiting the ability to consistently label specific cell types, especially in adult tissues. Thus, new genetic tools that reliably label cells by inserting reporter constructs at their native promoter sites would be of great interest.

Recent advances in genome editing that utilize sequence-specific DNA nucleases like Zinc finger nucleases, TALEN, and more importantly CRISPR/Cas9, have opened hitherto non-existent opportunities to knock-out or knock-in genes at precise locations in the zebrafish genome (Jao et al., 2013; Auer et al., 2014; Hoshijima et al., 2016). Double stranded breaks (DSB) created by the above-mentioned systems trigger cell repair mechanisms like the non-homologous end-joining (NHEJ) pathway that result in site-specific insertion/deletion (indel) mutations in the corresponding target sites. Recently, Auer et al. (2014) showed that reporter constructs could be efficiently integrated at the target site (TS) by providing a bait plasmid containing the TS, and have used this homology-independent knock-in system to convert GFP reporter lines into Gal4 driver lines or to directly knock-in Gal4 transgenes. Adopting this strategy, we have generated knock-in reporters by targeting them to a 5′ sequence upstream of the ATG for some of the essential genes involved in MHB development, namely, *Otx2* and *pax2a*. These transgenic lines act as read-outs for gene promoter activity and provide new tools to observe MHB development in real time.

## Materials and methods

### Zebrafish strains and maintenance

Zebrafish (*Danio rerio*) embryos were obtained by natural spawning. Both embryos and adults were raised and maintained at 28.5°C with a 14-h light and 10-h dark cycle (Westerfield, 2000; Brand et al., 2002). Embryos were staged as hours post fertilization (hpf) as described previously (Kimmel et al., 1995). The wild type strain AB was used to generate reporter knock-in lines, and transgenic fish lines were maintained as outcrosses. Neither the larvae nor the adult fish from the generated reporter lines showed any physiological or behavioral abnormalities.

This study was carried out in accordance with the animal welfare law (Tierschutzgesetz, Federal republic of Germany) and the local authority (Landesdirektion Sachsen). Protocols for the generation (24-9168.11-1/2013-14) and maintenance of transgenic animals (DD24-5131/346/11 and DD24-5131/346/12) were appropriately approved (Landesdirektion Sachsen). Experimental animals were used according to the approved protocols (24-9168.24-1/2014-4).

### Molecular cloning

Genomic DNA from the wild type strain AB was used to amplify bait sequences by PCR (Phusion Polymerase, Thermo Fischer) using primers listed in the Supplementary Table S2. To generate donor plasmids, baits were cloned into a pCS2+ or Topo PCR II vectors (Invitrogen) containing the coding sequence for the Venus fluorescent protein or turboRFP (promoter-less tRFP plasmid, Evrogen). The CMV promoter was later removed from the pCS2+ vector. All constructs were verified by sequencing.

### Single-guide RNA (sgRNA) design and off targets

The sgRNA sequences targeting the NGG-PAM motif were identified at about 500 base pairs upstream of the ATG start codon for a specific gene of interest. CHOP-CHOP web tool was used for selecting target sites (Montague et al., 2014). The sgRNA designed for *otx2* had the sequence GGAACccggCTAATTGTCTCAGG while that for *Pax2a* was GGGGggatctGGGAAGGAGGGGG; the PAM sequences are underlined. Loss of the restriction site was used to estimate the efficiency of the sgRNA (HpaII and XhoII respectively, marked in lowercase). No off-targets were identified for the chosen TS, using CHOP-CHOP i.e., there were no genomic targets with 2 bp mismatches (Cong et al., 2013). Further, the transgenic reporter lines have been outcrossed for more than 6 generations to date, thereby diluting out potential off target mutations.

### sgRNA, Cas9 generation, and injection into zebrafish embryos

The sgRNA and Cas9 mRNA were prepared as previously described (Jao et al., 2013), and plasmids for generating mRNA were a gift from the Chen and Wente labs (sourced from addgene). All injections were carried out in the wild-type AB strain embryo at the 1-cell stage. Each embryo was injected with a 1 nl solution containing 35 ng/μl of sgRNA, 150 ng/μl of Cas9 mRNA, and 25 ng/μl circular donor plasmid. The sgRNA and bait plasmid concentrations were optimized such that at least 50% of the injected embryos survived and showed normal development at 24 hpf. The injected embryos were monitored for the next 5 days and about 100 embryos were raised to adulthood for each transgenic line.

### Identification of founders and genotyping

Injected embryos were raised and outcrossed with the wild type strains WIK or TL. Founders were identified by screening F1 embryos for the presence of a fluorescence signal at 24 hpf. The screening was stopped when 2 founders were identified for each transgenic line. Genomic DNA was isolated from individual F1 embryos, and PCR amplification and subsequent sequencing were used to verify integration at the 5′ and 3′ junctions. F1 embryos with a fluorescence signal were raised and outcrossed to different wild-type strains (AB, WIK, or TL) between generations to reduce the general effects of inbreeding.

### RNA extraction and quantitative real time PCR (qRT PCR)

For both *otx2* and *pax2a*, the Venus knock-in fish were crossed with the tRFP knock-in line and the double-positive (Venus^+^ and tRFP^+^) embryos were sorted at 48 hpf. Respective wild-type control embryos (double-negative) were also collected at 48 hpf. The embryos were pooled (*n* = 15), lysed in extrazol (BLIRT S.A.), RNA extracted, and treated with DNAse. One-step real time reverse transcription PCR (Takara) was performed on biological (*n* = 3) and technical (*n* = 3) replicates to quantify expression of *otx2* and *pax2a* in the double-positive transgenic embryos (Venus^+^ and tRFP^+^) and was compared with that of wild-type embryos. Beta-actin was used as a house keeping gene to normalize the expression values. Fold changes were calculated using the 2^−ΔΔC^_T_ Method (Livak and Schmittgen, 2001) and the two tailed, unpaired “*t*”-test was used to calculate statistical significance at a “*p*”-value of 0.05 (Graph pad prism, ver. 5.0).

### Tissue preparation

Embryos (24 hpf) were fixed with 4% paraformaldehyde (PFA) and stored in 100% methanol at −20°C. For adult fish, fish aged between 6 and 8 months were killed by an MS-222 overdose and the heads harvested after carefully removing the skull roof. Fish heads were fixed overnight in freshly prepared 4% PFA in 0.1 M phosphate buffer (PB), pH 7.4. Fixed samples were subjected to decalcification overnight in PB containing 0.5 M EDTA and 20% sucrose prior to embedding in 7.5% gelatin and 20% sucrose in PB. Next, fish heads were instantly frozen on dry ice and cryo-sectioned at 7–10 μm thicknesses on a Microm HM 560 cryostat. Cryopreserved heads were stored at -80°C and the cryo-sectioned slides were stored at −20°C for subsequent immunohistochemistry (IHC).

### *In situ* hybridization

Embryos (24 hpf) were fixed in 4% PFA and stored in 100% methanol at −20°C. Whole mount *in situ* hybridization was performed as previously described (Reifers et al., 1998). Briefly, digoxigenin (DIG) or fluorescein-labeled probes, synthesized from linear DNA using a RNA labeling and detection kit (Roche), and hybridized probes were detected using anti-digoxigenin or anti-fluorescein antibodies. Antibody staining was visualized using BM purple (digoxigenin) or fast red (fluorescein). The stained embryos were dissected with sharpened tungsten needles, thick sections mounted in glycerol, and sections imaged in a Zeiss Axioplan microscope. *In situ* probe staining matched previously described expression for both *otx2* (Mercier et al., 1995) and *pax2a* (Krauss et al., 1991). For adult zebrafish brain sections, freeze-thawed and air-dried sections were treated with 100% Methanol (500 μl/slide) for 10 min, washed in PBS-TritonX100 (PBS-Tx) buffer prior to incubation with *in situ* probe (1:100 dilution), and denatured at 70°C in hybridization buffer. Hybridization was done overnight at 60°C in a humidified chamber. Excess/unbound probe was removed by rigorous washing with 1x SSC/50% formamide solution at 62°C. Sections were then washed with maleic acid buffer containing Tween-20 (MABT) at room temperature (RT). Sections were blocked in DIG blocking reagent (Roche) prior to anti-DIG antibody (1:2,000) incubation overnight at 4°C followed by washes with MABT solution to remove excess antibody. Sections were treated with NBT/BCIP diluted in staining buffer (1:17, NTMT) and the reaction was developed at RT until a signal appeared on the sections. Staining reaction was stopped by washing with PBS and slides were mounted with 80% glycerol. Images were acquired on a Zeiss Apotome using a differential interference contrast (DIC) filter and processed using ZEN Blue (ver. 2.3), Adobe Photoshop, and Adobe Illustrator (ver. CS5 and CS6) software.

### Immunohistochemistry and imaging

General immunohistochemistry (IHC) procedure was followed as described elsewhere (MacDonald, 1999). For IHC, slides were thawed from −20°C, air-dried, and washed twice with 1x PBS-Tx, 10 min each time. Primary antibodies were diluted in PBS-Tx and incubated overnight at 4°C and secondary antibodies were similarly diluted but incubated for 2 h at RT. The primary antibodies used were: polyclonal chicken anti-GFP (1:2,000, Abcam, Cat.No: ab13970) to detect Venus and monoclonal mouse anti-HuC/D (1:150, Invitrogen, Cat.No: A21271), and corresponding Alexa conjugated (488 or 555), highly cross-adsorbed, secondary antibodies (1:750, Invitrogen) were used for detection of primary antibodies. DAPI (4′, 6-diamidino-2-phenylindole; 1:3,000) was used to stain nuclei. Antigen retrieval for HuC/D was performed using 10 mM sodium citrate buffer, pH 6.0, at 85°C for 15 min. Immunostained samples were imaged on a laser scanning confocal microscope (Leica-SP5) using objectives 20x (0.7 NA), 40x (0.75 NA), or 63x Water (1.2 NA). Images were processed using Leica LAS X, Adobe Photoshop, and Adobe Illustrator (CS5 and CS6) software.

### Live imaging

Embryos were treated from 20 to 24 hpf with 1-Pheny 2-thiourea (PTU) to block pigmentation and with MS-222 for anesthesia, mounted on a glass bottom dish (MatTek) in 1% low melting agarose, and imaged on a Zeiss LSM 780 or Leica SP5 microscope. Images were analyzed using FIJI (open source software) or Imaris (ver. 7, Bitplane), respective TIFF files generated, and figures assembled in Adobe Photoshop (ver. CS5 or CS6). For time-lapse imaging, tissue sections spanning 30 μm, with a Z interval of 2 μm, were imaged every 8 min for about 6 h at 28°C on LSM780 (Zeiss) microscopes. Maximum intensity projections of fluorescence and transmitted light images were generated using Imaris (ver. 7, Bitplane).

## Results

### Targeted knock-in at the *otx2* locus to generate reporter lines

**Figure 1:**
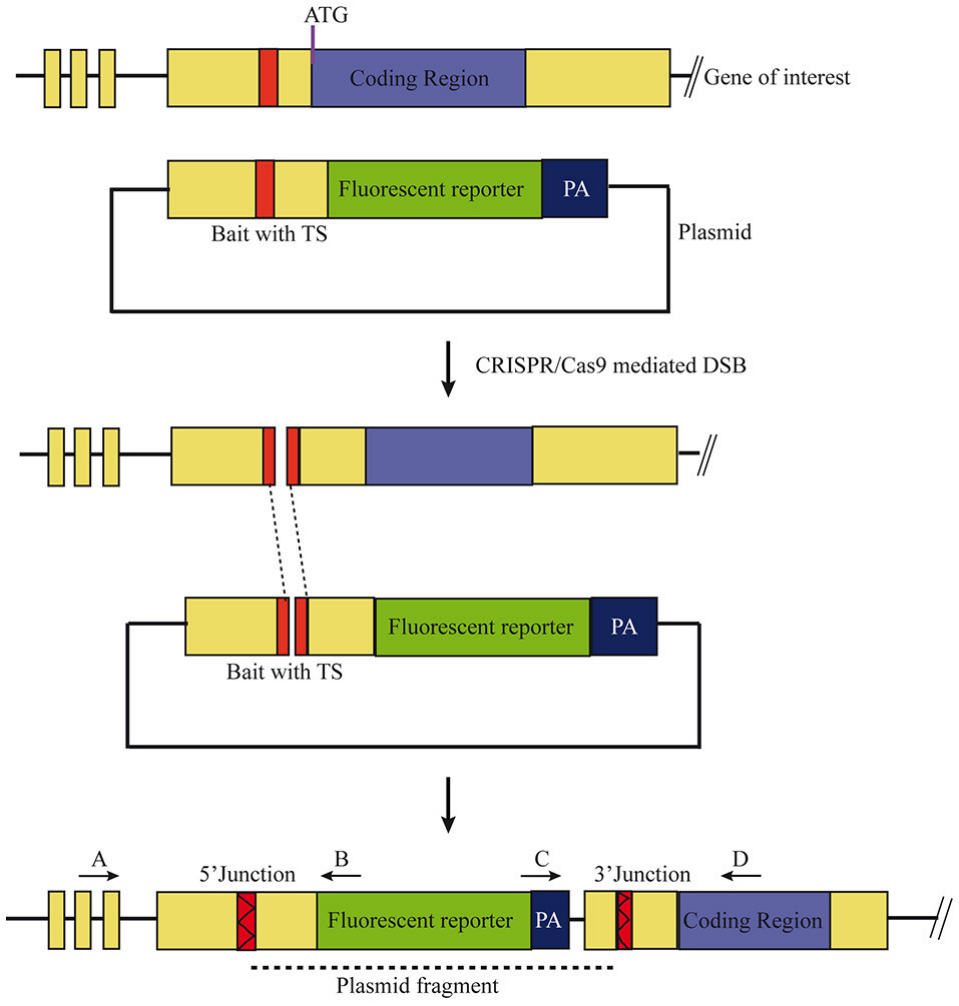
Scheme of the knock-in strategy. A target site (TS), located ~500 base pairs upstream of the ATG in the non-coding region in the gene of interest were chosen. The bait plasmid was constructed by cloning 1 kb of the upstream sequence, including the target site, into a plasmid with the desired fluorescent reporter and poly A (PA) sequence. The bait plasmid, sgRNA against the target site, and Cas9 mRNA were injected at the 1-cell stage. The Cas9 protein creates double stranded breaks (DSB) at both TS, i.e., genomic locus and bait plasmid, and the linearized plasmid bait is integrated by homology independent repair. Forward integration of bait plasmid will result in expression of the fluorescent reporter that matches the expression pattern of the gene of interest. Primer pairs (A+B, C+D) can be used to verify the 5′ and 3′ junctions of the knock-in, respectively.

**Figure 2:**
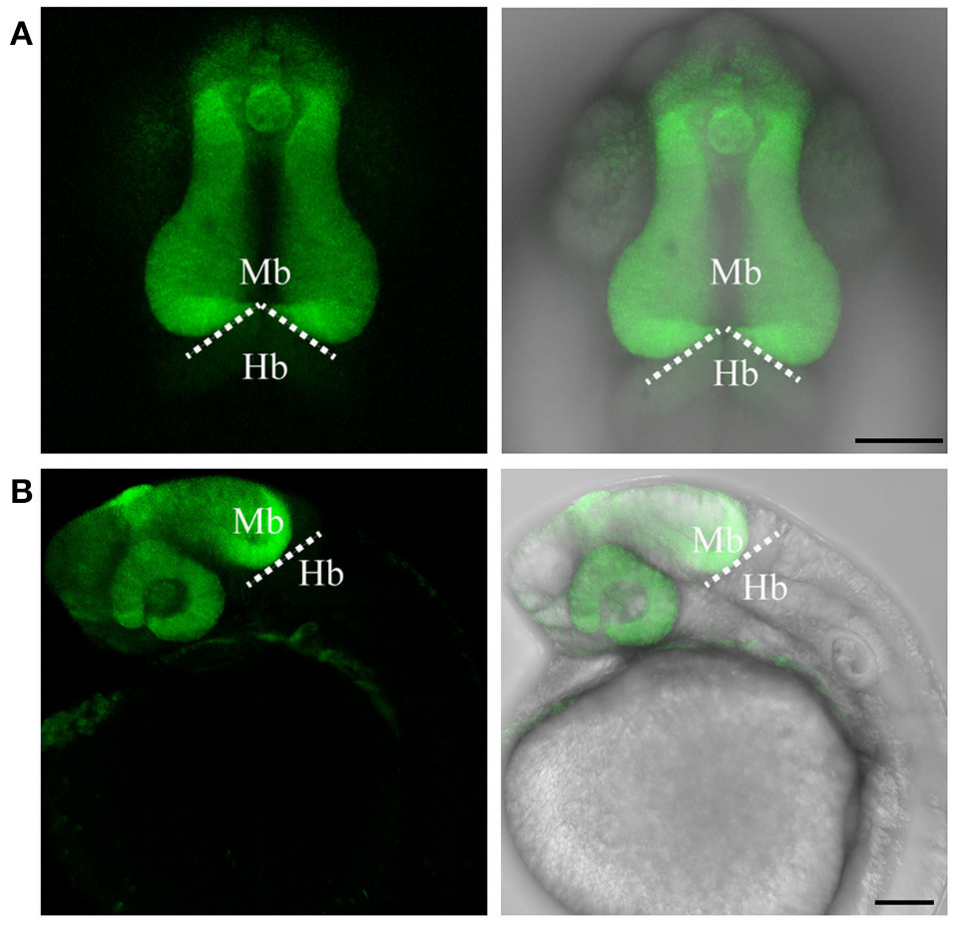
Targeted knock-in of Venus fluorescent protein into the *otx2* locus. Expression of Venus fluorescent protein at 24 hpf. Images were taken from live embryos anesthetized in MS-222. **(A)** Left panel shows dorsal view with the anterior of the embryo facing upwards, right panel is a merged image of the fluorescent and transmitted light channels. Venus is expressed in the retina and midbrain, sharply abutting the MHB (dotted line). **(B)** Lateral view of an embryo expressing Venus. Left panel shows fluorescent channel and right panel shows a merged image of the fluorescent and transmitted light channels. All images are maximum intensity projections covering 50 μm tissue with a Z-interval of 2 μm. Mb, midbrain; Hb, hindbrain. Scale bar: 100 μm.

**Figure 3:**
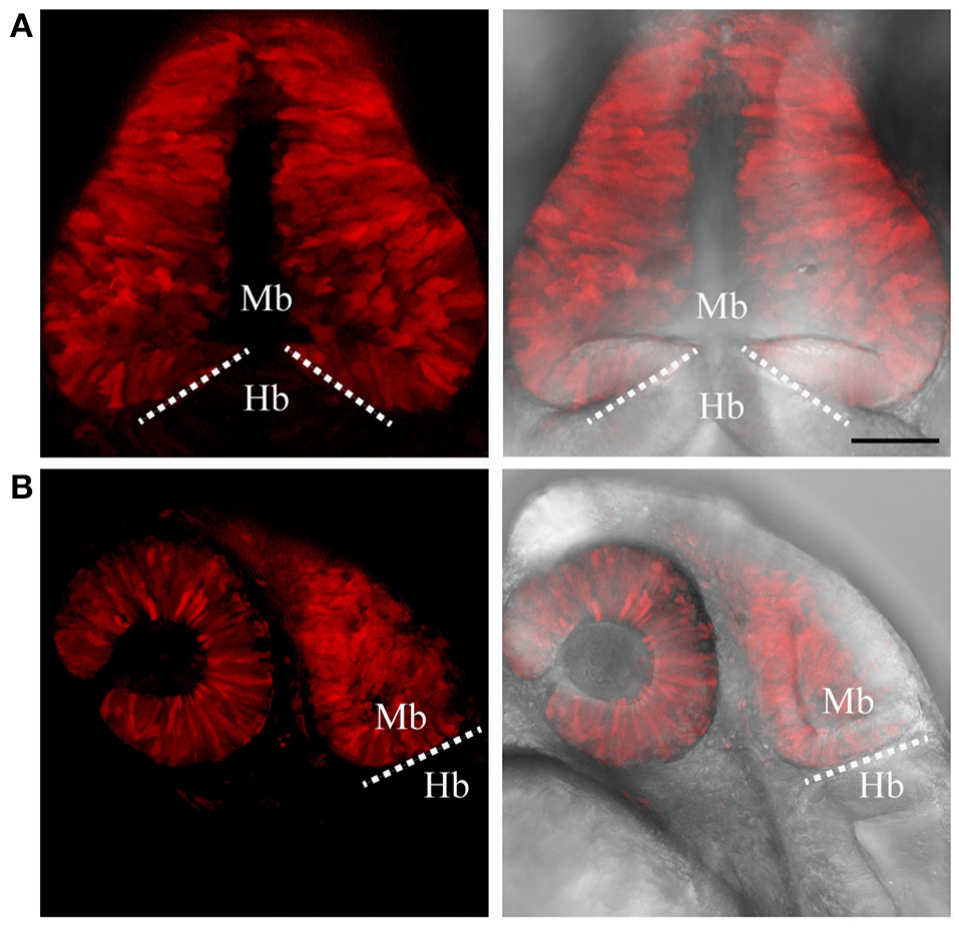
Targeted knock-in of tRFP into the *otx2* locus. Expression of tRFP at 24 hpf. Images were taken from live embryos anesthetized in MS-222. **(A)** Left panel shows a dorsal view with the anterior of the embryo facing upwards, right panel shows a merged image of fluorescent and transmitted light channels. tRFP is expressed in the retina and midbrain, sharply abutting the MHB (dotted line). **(B)** Lateral view of embryos expressing tRFP. Left panel shows fluorescent channel and right panel shows a merged image of the fluorescent and transmitted light channels. Images are maximum intensity projections covering 50 μm tissue with a Z-interval of 2 μm. MB, midbrain; Hb, hindbrain. Scale bar: 100 μm.

**Figure 4:**
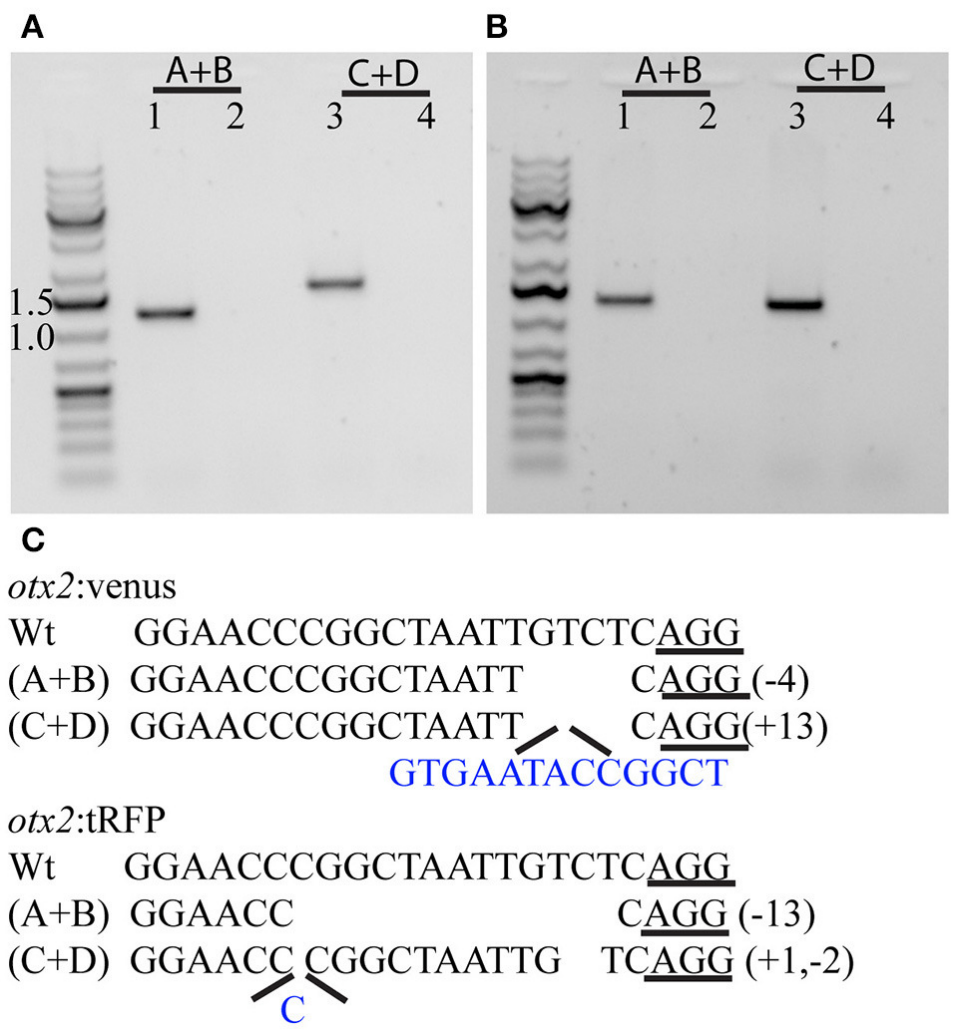
Characterization of the 5′ and 3′ junctions of the knock-in at the *otx2* locus. Representative gel pictures of the *otx2*:venus **(A)** and *otx2*:tRFP **(B)** knock-in alleles from one founder (lanes 1 and 3) or wild-type siblings (lanes 2 and 4). Lanes 1 and 2 show the 5′ junction PCR (primer pair A+B from Figure 1) and lanes 3 and 4 show the 3′ junction (primers C+D from Figure 1). **(C)** DNA sequence analysis of the 5′ and 3′ junctions and mutations; “−” denotes deletion and “+” denotes insertion; inserted base pairs are marked in blue.

To test if the CRISPR/Cas9-mediated strategy can be used for knocking-in reporters into the zebrafish genome, we chose *otx2* as a candidate gene because, first the caudal limit of the Otx2 marks the MHB, and second, no transgenic *otx2* reporter lines that recapitulate its endogenous expression are currently available (Kurokawa et al., 2006). Fast maturing fluorescent proteins such as Venus and turboRFP (tRFP) were used as reporters. The target site (TS) was selected at about 500 base pairs upstream of the transcription start site of *otx2*, and about 1 kb of bait that included the target region was amplified and cloned in front of the fluorescent reporter (Figure 1, scheme). The target site sequence was verified prior to cloning by DNA sequencing. The efficiency of target-site-cutting was assayed as follows. Both sgRNA and Cas9 were injected into the 1-cell stage embryo, and genomic DNA from individual 24 hpf embryos was isolated for PCR amplification. Loss of the restriction site was used to estimate the efficiency of the sgRNA, and those with >50% efficiency were selected (data not shown). Next, the bait plasmid was injected along with the sgRNA and Cas9, and concurrent double strand breaks were generated in the genomic target locus and in the plasmid DNA, resulting in plasmid integration at the target locus, most probably due to the highly active non-homologous end enjoining (NHEJ) repair mechanism of the cells. To identify founders, adult F0 fish were outcrossed with wild-type animals and F1 embryos were screened for fluorescence. Positive embryos and a representative embryo at 24 hfp are shown (Figures 2A,B, 3A,B). Reporter expression perfectly matched the expected expression pattern of Otx2 (Langenberg and Brand, 2005) as a sharp boundary was observed at the MHB, with no ectopic fluorescence in the non-Otx2^+^ regions. Two founders were identified for each construct with no difference in reporter gene expression pattern among them (data not shown). The knock-in was verified by PCR using primers designed such that the forward primer annealed at the expected knock-in genomic locus (but outside the bait sequence) and the reverse primer within the fluorescent reporter donor plasmid (Figures 4A,B). Sanger DNA sequencing confirmed the knock-in location and showed indels at the 5′ and 3′ integration site (Figure 4C). Germline transmission rates of successful founders were 4% for *otx2*-Venus and 7.6% for *otx2*-tRFP reporter lines (summarized in Supplementary Table S1). Currently, *otx2* reporters have been outcrossed for more than six generations and transgene expression has remained stable (data not shown). Further, such outcrossing of the transgenic reporters with wild-type strains over several generations would dilute out any potential off target mutations.

### Knock-in alleles remain functional

**Figure 5:**
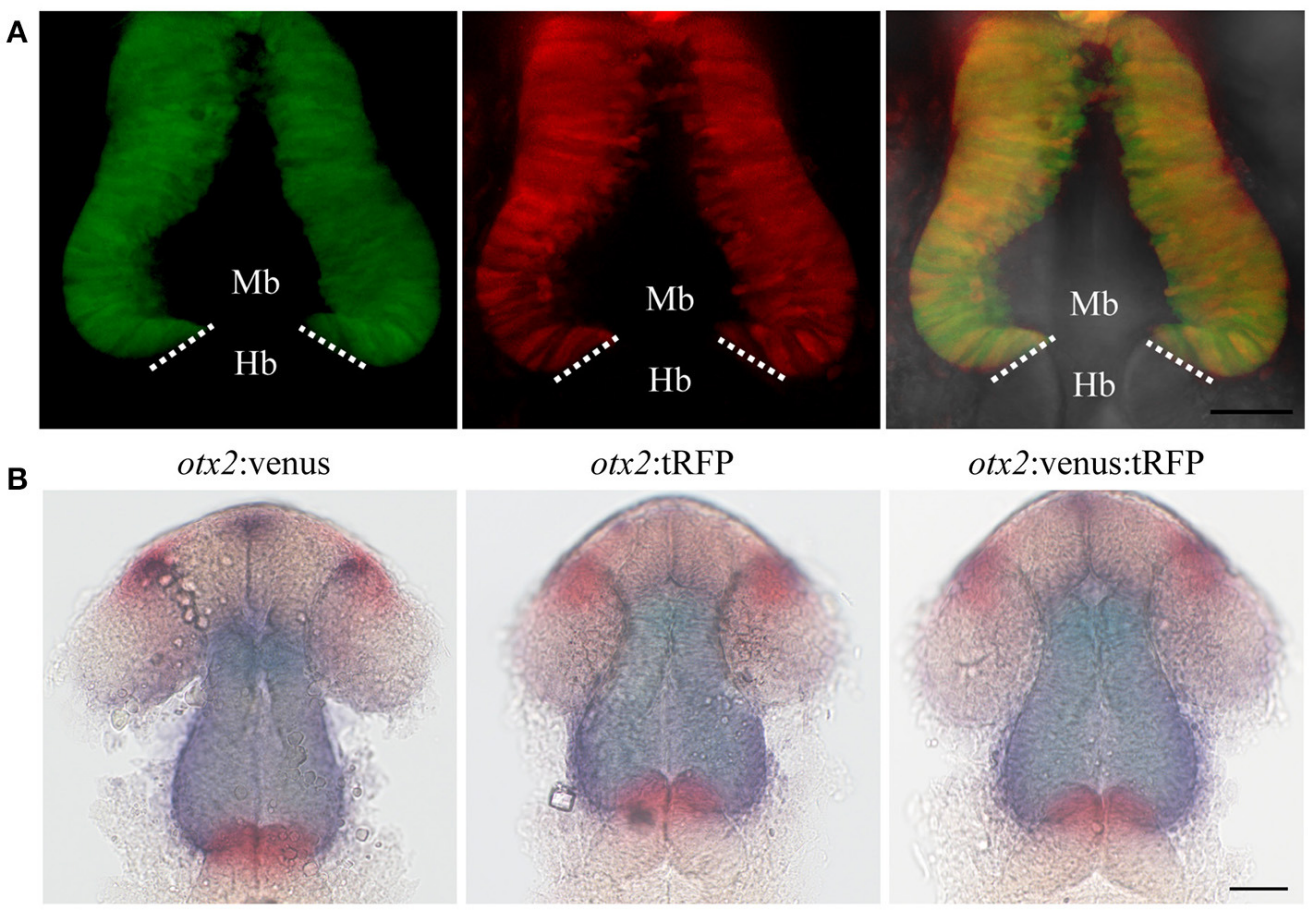
Reporter knock-in into the *otx2* locus does not affect endogenous *otx2* expression. *otx2*:venus and *otx2*:tRFP fish were crossed and sorted into either single (Venus^+^ or tRFP^+^) or double positive (Venus^+^ and tRFP^+^) embryos at 24 hpf. Images were taken from live embryos anesthetized in MS-222. Panel **(A)** fluorescent images of the single- and double-positive embryos show no morphological abnormalities. Images are maximum intensity projections covering 50 μm tissue with a Z-interval of 2 μm. Scale bar: 50 μm. Images were taken from live embryos anesthetized in MS-222. Panel **(B)** Double *in situ* hybridization for *otx2* (blue) and the MHB marker *pax2a* (red) show no differences in gene expression pattern or morphology of the MHB region between the single- and double-positive groups. Mb, midbrain; Hb, hindbrain. Scale bar 50 μm.

Next, we addressed if the knock-in reporters that include a polyA signal and the vector backbone sequences at the 5′ upstream of ATG compromise endogenous gene expression. Thus, we crossed the Venus knock-in fish with the tRFP knock-in line and sorted embryos at 24 hpf into either single-positive (Venus^+^ or tRFP^+^) or double-positive (Venus^+^ and tRFP^+^) embryos. Morphological examination showed no differences between the single- and double-positive groups (Figure 5A); subsequent analysis of MHB morphology and double *in situ* hybridization for *otx2* and the MHB marker *pax2a* also showed no differences (Figure 5B). Further, quantitative real time PCR (qRT-PCR) showed no significant difference in *otx2* expression levels in the double positive embryos compared to wild-type (**Figure 8D**). These data suggest that reporter genes can be efficiently knocked-in at the *otx2* locus, and that integration of a reporter plasmid into the non-coding region, upstream of ATG, does not interfere with endogenous gene expression or function.

### Practical application: live imaging MHB development

**Figure 6:**
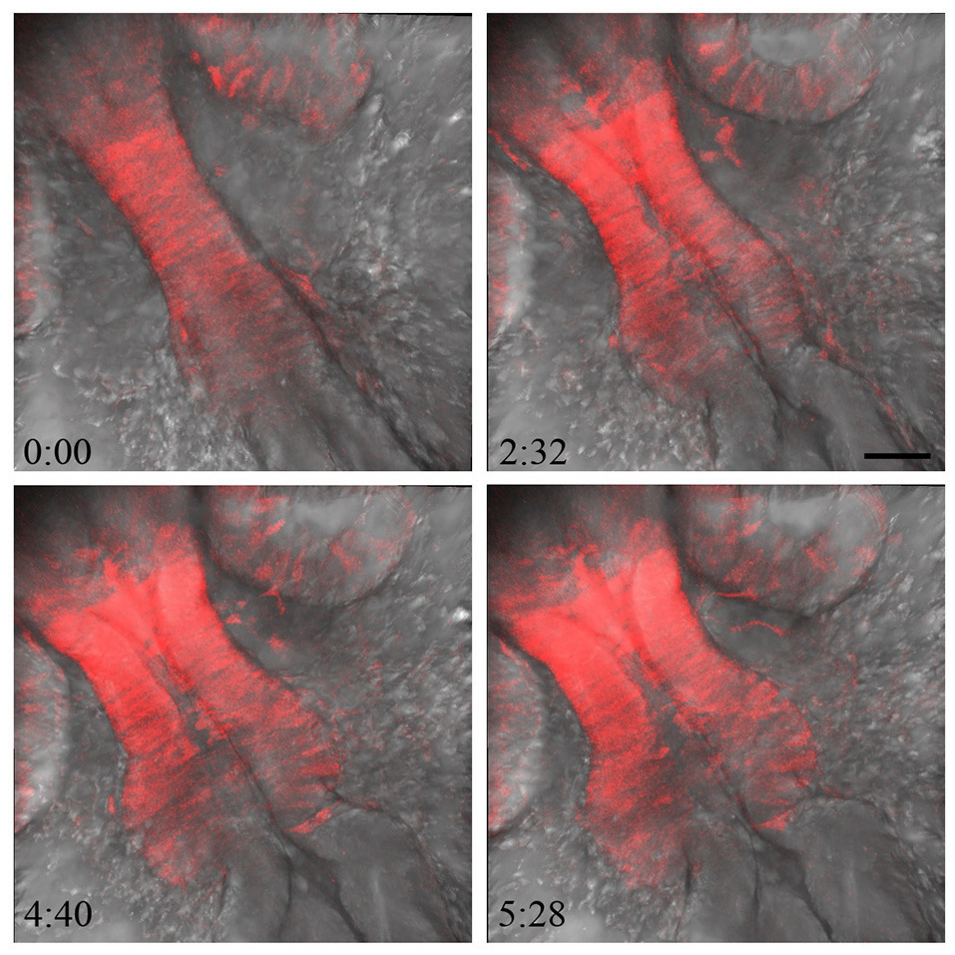
Imaging MHB development in real time. To visualize MHB development in real time, embryos from the *otx2*:tRFP reporter line were mounted and imaged dorsally from 17 to 22 hpf. Tissue sections covering about 40 μm were chosen with a z interval of 2 μm. Images were acquired at 6 min intervals. The *otx2*:tRFP + cells in the midbrain are clearly visible and cell movements can be followed during ventricular space opening and folding of the neural tube at the MHB. In addition, cellular rearrangements in delaminating neural crest cells and retinal cells are also visible. Time in minutes: seconds. Scale bar: 50 μm.

Neural tube formation and the underlying cell-biology processes that generate the 3 dimensional structure of the vertebrate brain are of great interest. Hence, real time or time-lapse imaging to understand these morphogenetic processes is a valuable approach. We utilized the reporter lines generated using the CRISPR/Cas9 system to observe MHB development in real time. The *otx2*:tRFP reporter line was used to follow midbrain cells during MHB formation between 17 and 23 hpf, when the opening of the ventricular space and constriction at the MHB occur. In addition, *otx2*:tRFP^+^ neural crest cells leaving the neural tube, as well as the *otx2*:tRFP^+^ cells in the retina, can be observed (Figure 6 and Supplementary Movie 1). Consequentially, the ability to co-label cell membranes and nuclei will be valuable in further elucidating the morphogenetic processes that occur during MHB development, including cell shape changes, cell division, and acquisition of apical-basal polarity.

### Targeted knock-in at the *pax2a* locus

**Figure 7:**
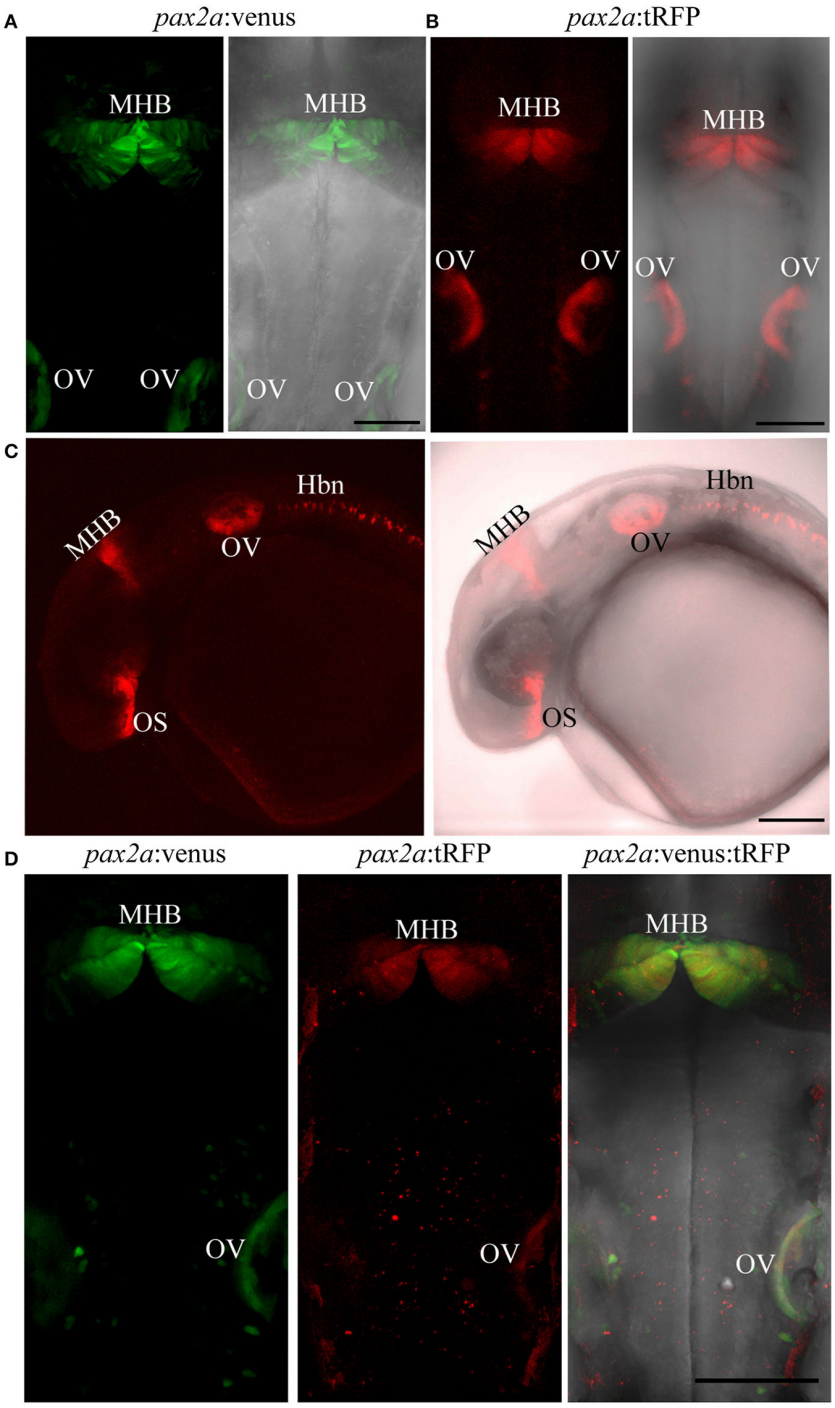
Targeted knock-in of fluorescent reporters into the *pax2a* locus. Expression of Venus or tRFP driven by the *pax2a* locus at 24 hpf. Images were taken from live embryos anesthetized in MS-222. **(A)** Left panel shows dorsal view of Venus expression in the MHB and otic vesicle (OV); right panel shows merged image of fluorescent and transmitted light channels. **(B)** Left panel shows dorsal view of tRFP expression in the MHB and otic vesicle. **(C)** Left panel: lateral view of an embryo expressing tRFP in the optic stalk (OS), MHB, OV, and hindbrain neurons (Hbn). Right panel: merged image of fluorescent and transmitted light channels. **(D)** *pax2a*:venus and *pax2a*:tRFP fish were crossed and resulting progeny was sorted into either single (Venus^+^ or tRFP^+^) or double positive (Venus^+^ and tRFP^+^) embryos at 24 hpf. Compared to single-positive siblings, double positive embryos show no morphological abnormalities. All images are maximum intensity projections covering 50 μm tissue with a Z-interval of 2 μm. Scale bar 100 μm.

**Figure 8:**
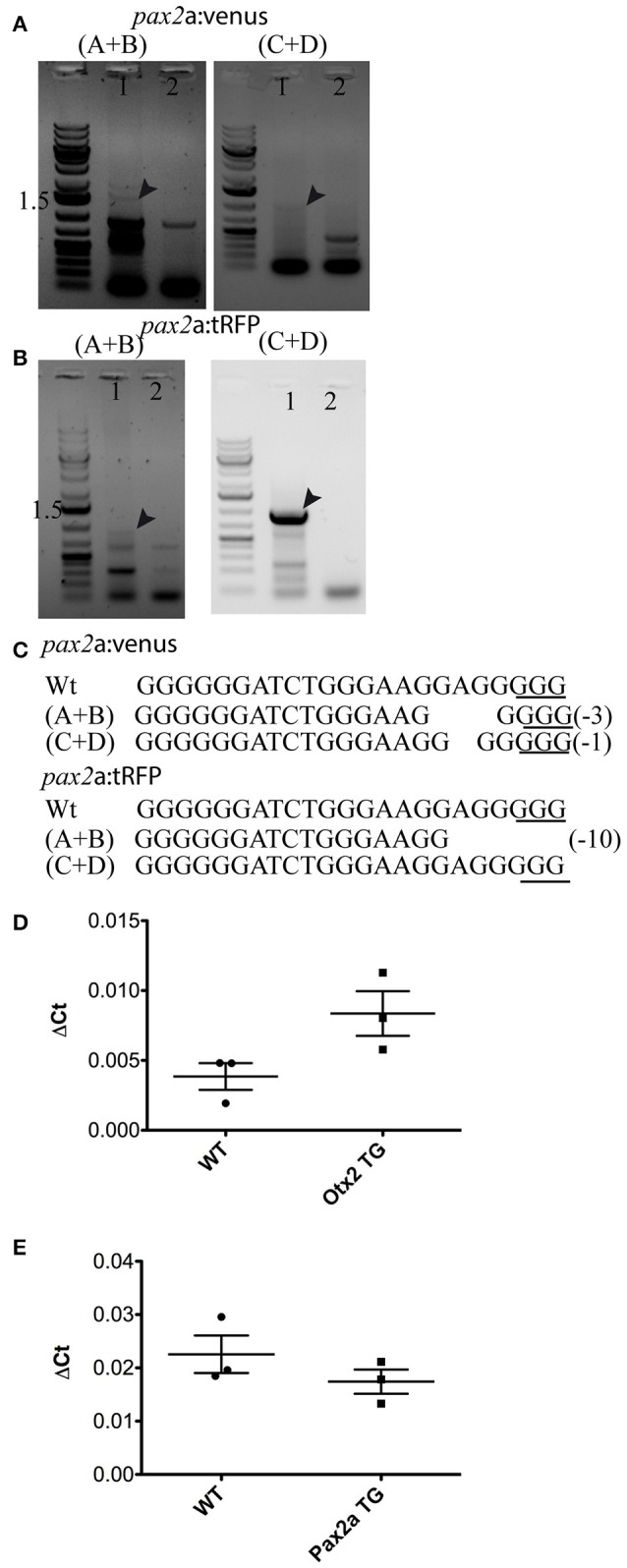
Validation of knock-in insertions at the *pax2a* locus and gene expression quantification. Representative gel pictures of the *pax2a*:venus **(A)** and *pax2a*:tRFP **(B)** knock-in alleles from one founder (lane 1) and wild-type siblings (lane 2). Left panel shows 5′ junction PCR (primer pair A+B from Figure 1) and the right panel shows 3′ junction (primers C+D from Figure 1). **(C)** DNA sequence analysis of the 5′ and 3′ junctions and mutations; “−” denotes deletion. **(D,E)** A comparison of gene expression between homozygous embryos (*otx2* or *pax2a* transgenic animals (TG) positive for both venus and tRFP) and wild-type (WT) embryos (48 hpf) showed no significant differences in the mRNA levels for *otx2* **(D)** and *pax2a* **(E)**. The two-tailed, unpaired *t*-test was used to calculate statistical significance; each point in the graph represents 1 sample, which contains a pool of 15 embryos. WT vs. *otx2, p* = 0.072, WT vs. pax2a, *p* = 0.288.

To test the knock-in strategy on other genomic loci, we selected the *pax2a* locus, as it is one of the earliest MHB markers. Currently, there are no *pax2a* reporter lines that recapitulate all its endogenous expression domains (Picker et al., 2002). Again, a highly efficient sgRNA that cleaves the target site upstream of ATG was chosen (data not shown) and bait plasmids were constructed with Venus or tRFP as fluorescent reporters. Embryos were injected and founders were identified as explained above. Fluorescence was evident in the optic stalk, the MHB, and in the optic vesicle at 24 hpf (Figures 7A–C); this expression pattern matches the reported expression pattern for *pax2a* (Krauss et al., 1991; Lun and Brand, 1998). Germline transmission rates for successful founders were 20% for *Pax2a*:venus and 2.8% for *Pax2a*:tRFP reporters (summarized in Supplementary Table S1). The knock-in was verified by PCR using primers designed such that the forward primer annealed at the expected knock-in genomic locus (but outside the bait sequence) and the reverse primer within the fluorescent reporter donor plasmid (Figures 8A,B). Sanger DNA sequencing confirmed the knock-in location and showed indels at the 5′ and 3′ integration site (Figure 8C). To test if the knock-in alleles still expressed endogenous *pax2a*, we crossed the Venus and tRFP reporter lines and found that the double positive embryos (Venus^+^ and tRFP^+^) were morphologically normal (Figure 7D) and the pax2a mRNA remain unchanged in the double positive embryos as quantified by qRT-PCR (Figure 8E). These data suggest that, similar to the *otx2* knock-in, the *pax2a* locus can also be targeted for genome editing and that the reporter knock-in does not interfere with endogenous gene function.

### *otx2*:venus labels neurons and the radial glia in the adult zebrafish midbrain

**Figure 9:**
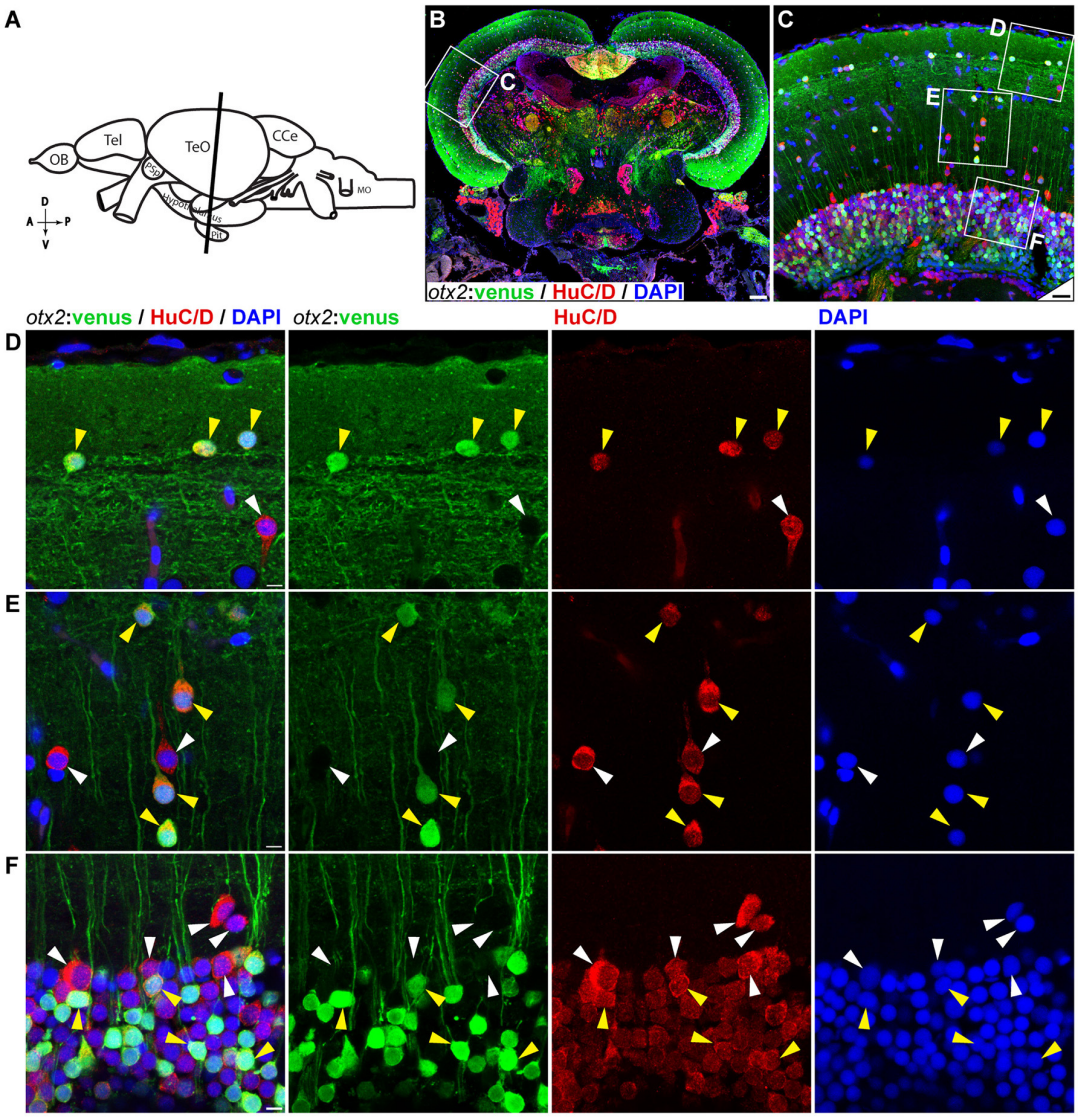
*otx2:*venus expression in the adult zebrafish midbrain. **(A)** Schematic representation of the adult zebrafish brain. The position of the cross section of the midbrain with optic tectum, hypothalamus, and pituitary shown in panel **(B)** is indicated (orientation indicators A->P: anterior to posterior; D->V: dorsal to ventral). **(B)** Cross section of the midbrain labeled with *otx2*:venus (green), the pan neuronal marker HuC/D (red) and the nuclear counterstain DAPI (blue). **(C)** Higher magnification image of the region indicated by white box in panel **(B)** showing that numerous neurons express *otx2*:venus. **(D–F)** Higher magnification images of the regions indicated by white boxes in **(C)**. *otx2*:venus is co-expressed in HuC/D expressing neurons (yellow arrowheads) in the various tectal sub layers that is characteristic of a teleost midbrain. However, a sub-population of HuC/D positive neurons was negative for *otx2*:venus (white arrowheads). **(D)** Type III horizontal neurons that belong to the stratum opticum. **(E)** Projection neurons that mostly belong to the stratum griseum et album superficiale and centrale regions. **(F)** Prominent unipolar type XIV interneurons located in the periventricular gray zone of optic tectum. Anatomical descriptions are based on the zebrafish brain atlas (Wullimann et al., 1996), tectal sub layers and neuronal types were interpreted based on Meek (1981) and Meek and Schellart (1978). Scale bars **(B)**: 100 μm; **(C)**: 20 μm; **(D–F)**: 5 μm. (**B,C**: maximum intensity projection; **D–F**: single Z-Plane).

**Figure 10:**
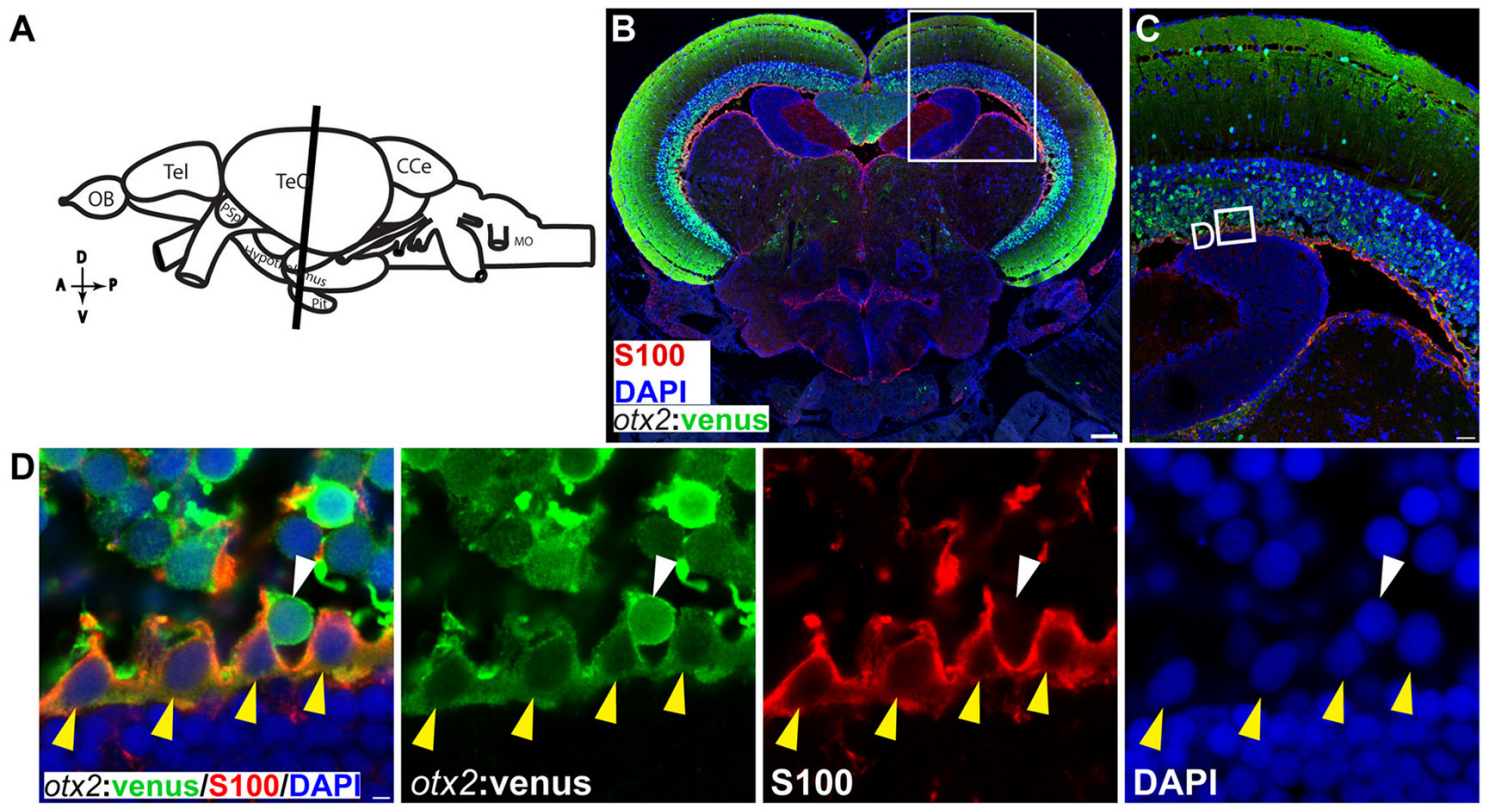
*otx2:*venus expression in radial glia located at the ventricular zone of the adult zebrafish midbrain. **(A)** Schematic representation of the adult zebrafish brain. The position of the cross section of the midbrain with optic tectum, hypothalamus, and pituitary shown in panel **(B)** is indicated (orientation indicators A->P: anterior to posterior; D->V: dorsal to ventral). **(B)** Cross section of the midbrain labeled with *otx2*:venus (green), the radial glial marker S100 (red) and the nuclear counterstain DAPI (blue). **(C)** Higher magnification of the region from midbrain tectum indicated by white boxes in **(B)**. **(D)** Higher magnification of an inset from panel **(C)** showing radial glial cells at the ventricular zone of the midbrain tectum labeled by S100 co-expressing *otx2*:venus. Anatomical descriptions are based on the zebrafish brain atlas (Wullimann et al., 1996). Scale bars **(B)**: 100 μm; **(C)**: 25 μm; **(D)**: 2 (or) 5 μm. (**B,C**: maximum intensity projection; **D**: single Z-plane).

**Figure 11:**
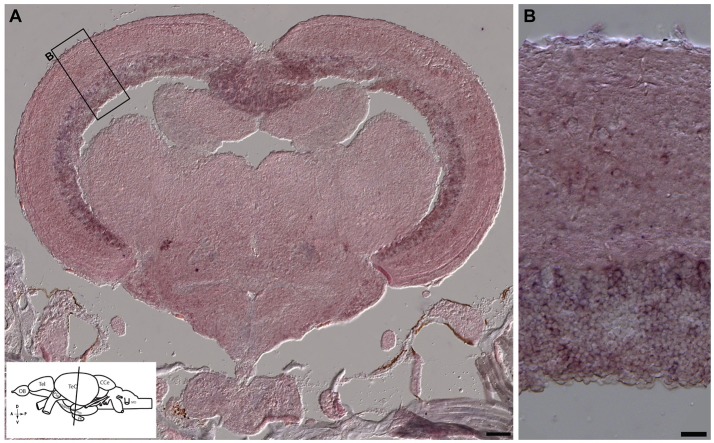
*In situ* hybridization showing *otx2* expression in the adult zebrafish midbrain. **(A,B)** *In situ* hybridization for *otx2* was carried out on coronal sections of the adult zebrafish midbrain (scheme shown in **A**). *Otx2* signal resembled reporter expression (Figures 8, 9) in the various tectal layers and particularly strong signals were observed in the periventricular gray zone of the optic tectum. A representative region is the marked with a rectangle and magnified in the adjacent panel **(B)**. Scale bars **(A)**: 100 μm; **(B)**: 20 μm.

We next tested the applicability of the *otx2*:venus reporter line in adult fish. Anatomically (Figure 9A, schematic), cells expressing Venus were prominently present in the neuronal nuclei or in the neurons of the midbrain, and specifically in the tectum opticum (TeO), the periventricular gray zone of the optic tectum, and the hypothalamus (Figure 9B). Co-expression analysis of Venus, with either HuC/D (a pan-neuronal marker) or S100β (a marker for glial cells), revealed that *otx2:venus* is expressed mostly in HuC/D^+^ neurons in the gray matter and in glial cells at the ventricular zone of the TeO (Figure 9C). Further, *otx2:*venus and HuC/D^+^ cells were present in various cortical layers of the tectum, such as the stratum opticum (Figure 9D), stratum griseum et album superficiale, and centrale (Figure 9E), and the periventricular gray zone (Figure 9F). However, not all HuC/D^+^ neurons were labeled by *otx2*:venus in the tectum, suggesting that Otx2 is expressed only in a sub-population of neurons and that this line might be a good tool to study this specific neuronal network. Based on previously described anatomical distribution and cell shape characteristics (Meek and Schellart, 1978; Meek, 1981), Venus^+^ cells appear to be type III horizontal neurons that belong to the stratum opticum (Figure 9D), projection neurons of the cortical layers stratum griseum et album superficiale and centrale (Figure 9E), and XIV interneurons of the periventricular gray zone (Figure 9F). Apart from this, *otx2*:venus also labeled S100β^+^ glial cells in the ventricular zone (anatomical location schematized in Figure 10A, overview images Figures 10B,C). S100β^+^ cells are seen at the ventricular zone co-expressing *otx2*:venus (yellow arrowheads) while the neighboring *otx2*:venus^+^ neurons are negative for S100β (Figure 10D). Most importantly, in the adult midbrain, *otx2:*venus expression mirrored endogenous *otx2* mRNA expression in the various tectal layers, as shown by *in situ* hybridization (Figures 11A,B). Taken together, *otx2:venus* CRISPR/Cas9 knock-in line appears to label various neuronal and radial glial populations in the adult zebrafish midbrain. The observed selective labeling presents a useful tool to study specific cells of interest while simultaneously overcoming the potential silencing effects that are often observed with other transgenic approaches.

### *pax2a*:venus reporter expression in the adult zebrafish midbrain

**Figure 12:**
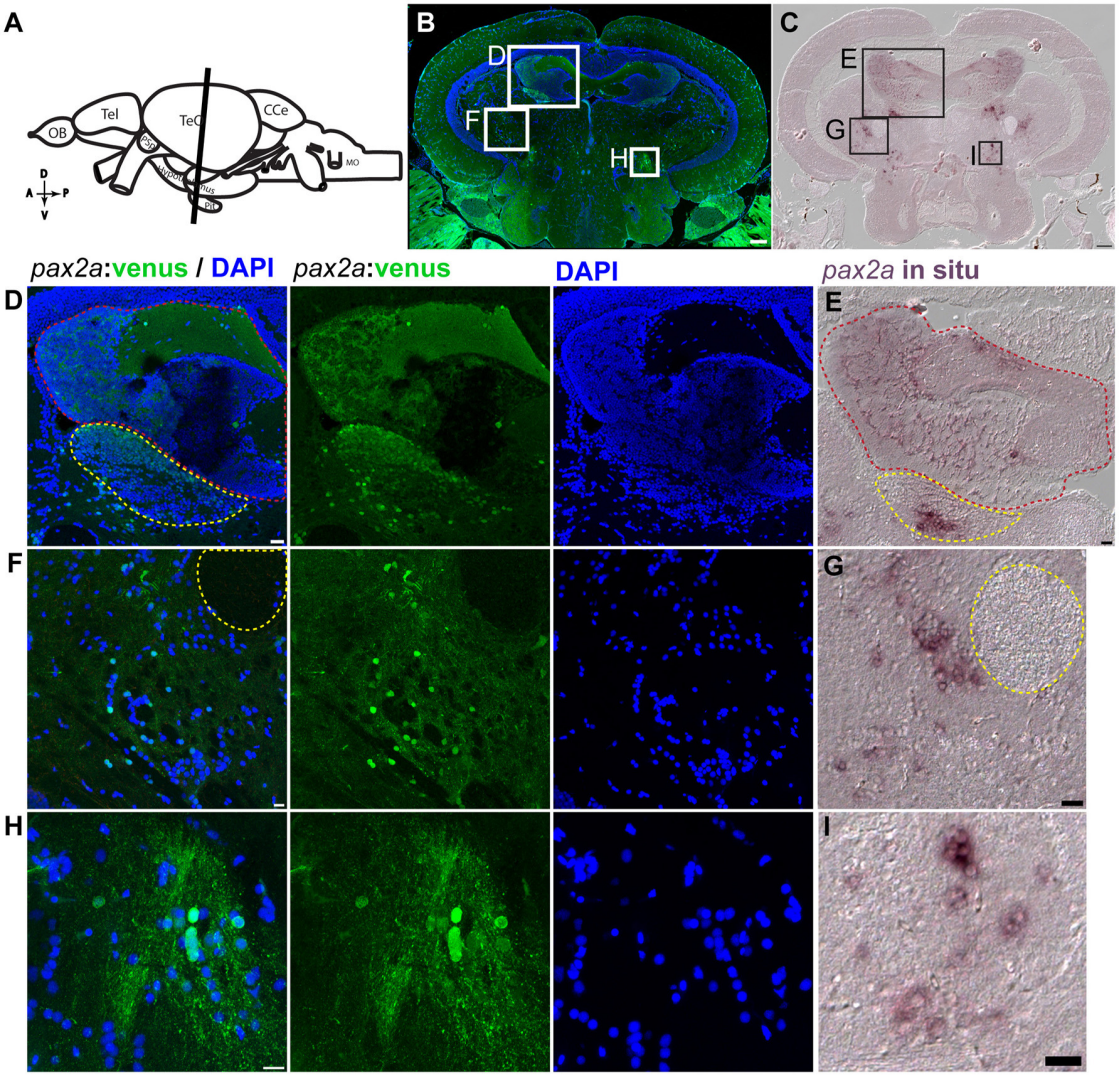
*pax2a:*venus expression in the adult zebrafish midbrain. **(A)** Scheme indicating a plane of coronal section of the adult zebrafish mid brain with optic tectum, hypothalamus and the pituitary corresponding to the cross section in “**B**” (orientation indicators A->P: anterior to posterior; D->V: Dorsal to Ventral). **(B)** Cross section of the midbrain immunostained against venus (green) and DAPI (blue). **(C)** *in situ* hybridization against pax2a in the midbrain similar to the coronal planes in “**A,B**” showing similar expression pattern as that of venus in the knock in reporter line. **(D,F,H)** Higher magnification of a midbrain indicated by white boxes in “**B**” showing neuronal nuclei expressing venus in regions, valvula cerebelli (red dotted line area) and dorsal tegmental nucleus (yellow dotted line area) “**D**,” neighboring (left side) to the lateral longitudinal fascicle “**F**” and the rostral tegmental nucleus “**H**.” **(E,G,H)** Higher magnification of a region from the midbrain tectum indicated by black boxes in “**C**” showing *in situ* expression pattern for pax2a showing highly similar pattern to their counter parts expressing *pax2a*:venus. Anatomical descriptions were based on (zebrafish brain atlas; Wullimann et al., 1996). Scale bars **(B,C)**: 100 μm; **(D,E,G,I)**: 20 μm; **(F,H)**: 10 μm. (**B**: max intensity projection; **D,F,H**: single Z-Plane; **C,E,G,I**: DIC image).

The *pax2a:*venus reporter labeled several neuronal subpopulations (anatomical location scheme Figure 12A, overview images Figure 12B). Most importantly, in the adult midbrain, *pax2a:*venus expression mirrored endogenous *pax2a* mRNA expression, as shown by *in situ* hybridization (Figures 12B,C). Essentially, *pax2a:*venus expression was prominently seen in the neurons of the valvula cerebelli in the hindbrain (red dotted line), the dorsal tegmental nucleus of the midbrain (yellow dotted line area; Figures 12D,E), and other regions of the midbrain. Based on previous anatomical descriptions of the zebrafish brain (Wullimann et al., 1996), *pax2a:*venus cells could be mapped to neurons present adjacent (left side) to the lateral longitudinal fascicle (yellow dotted circle), with some of these cells possibly belonging to the perilemniscal nucleus (Figures 12F,G). The rostral tegmental nucleus, another neural nucleus, was also positive for *pax2a*:venus expression (Figures 12H,I). However, there were no Venus+ cells in the glial domain. These results clearly indicate that *pax2a*:venus, similar to *otx2*:venus, labels a subpopulation of neurons in the mid- and hindbrain in the adult zebrafish.

## Discussion

Here we demonstrate that fluorescent reporters can be efficiently knocked into a specific locus for a gene of interest using the CRISPR/Cas9 system as a genome-editing tool. We have generated four knock-in reporter lines targeting two essential genes involved in MHB development, namely *otx2* and *pax2a*. The exact mechanism of integration (knock-in) at the target site has not been explored in this study. However, given that NHEJ has a greater probability of occurrence in zebrafish (Hagmann et al., 1998; Dai et al., 2010) and homologous recombination (HR) of 5 kb or larger plasmids has not yet been demonstrated, we think that NHEJ is the most probable mechanism of integration. Other studies in zebrafish using similar strategies have also found homology independent mechanisms such as NHEJ to be the primary mechanism that drives integration (Auer et al., 2014; Kimura et al., 2014; Ota et al., 2016).

The combination of methods used here, i.e., CRISPR and non-homologous end joining, has several advantages over BAC or promoter fragment-driven transgenesis. First, by knocking-in the reporter just in front of the endogenous ATG, we utilize the native promoter and enhancer elements that regulate endogenous gene expression. Consequently, reporter expression essentially recapitulates native/endogenous promoter activity and gene expression patterns. Indeed, we show that both Venus and tRFP reporter expression precisely match endogenous gene expression of *otx2* and *pax2a*, both in embryos and adults. The *Tg(pax2a:GFP)*^*e*1*Tg*^ transgenic line that we previously generated (Picker et al., 2002) used native promoter/enhancer elements that recapitulated parts of the endogenous *pax2a* expression pattern. However, this line also showed ectopic GFP expression in the forebrain, and in rhombomeres 3 and 5 of the hindbrain. Attempts to establish a stable transgenic zebrafish line that recapitulated *otx2* expression using combinations of various promoter/enhancer elements have failed (Kurokawa et al., 2006). Importantly, the knock-in reporter lines reported here also mirror endogenous gene expression patterns in the adult zebrafish brain, as defined by *in situ* hybridization. This aspect is valuable during both embryonic development and for identifying different neuronal and non-neuronal subtypes in the adult zebrafish brain. To our knowledge, this is the first study on reporter expression for *otx2* and *pax2a* in the adult zebrafish brain. Further applications of these reporter lines include their use to better understand the molecular characteristics of Otx2^+^ and Pax2a^+^ cells during different developmental and adult stages by subjecting them to fluorescence activated cell sorting (FACS) for transcriptomic and/or proteomic analyses.

Second, reporter knock-in at the non-coding region, just in front of the ATG did not negatively affect or nullify endogenous gene expression and is consistent with normal MHB morphology in the double transgenic reporters (Venus and tRFP positive). In contrast, recently, Ota et al. (2016) have reported a similar strategy to knock-in eGFP at the same genomic locus for *pax2a*, but the knocked-in allele generated a *pax2a* mutant allele resembling the homozygous *pax2a* null allele, *no isthmus* (*noi*; Brand et al., 1996). Differences in bait construction might account for why our strategy did not result in null alleles, compared to Ota et al. (2016). Specifically, in our strategy, the plasmid bait supplies the 500 base pairs of the *pax2a* 5′ sequence in front of the ATG, whereas Ota et al. (2016) used a universal bait sequence containing *hsp70l* promoter/enhancer elements and eGFP. Thus, our knock-in strategy can also be applied for generating Cre- or Gal4-driver lines that can be subsequently used for lineage tracing and loss-of-function studies. Recently, Suzuki et al. have reported the use of mini circles for highly efficient, homology-independent, targeted, *in vivo* integration. Using this method, they avoid plasmid backbone co-integration and their design of the targeting construct favors forward integration (Suzuki et al., 2016). The use of mini circles could be easily adapted to improve our strategy as it will avoid plasmid backbone integration, and the smaller size of mini circles may then improve targeting efficiency.

Third, the relatively high germ line transmission rates (ranging from 2.8 to 20%) will facilitate targeting of several gene loci as potential reporter lines. Hence, easy generation of Cre/Gal4-based driver lines is feasible because the target site resides in the non-coding region, making screening for in-frame insertions unnecessary. It is important to mention here that fluorescent protein expression is indeed a read-out of the promoter activity. It is for this reason that we chose fast folding proteins like Venus and tRFP as reporters, so that positive cells are labeled rapidly after promoter activation. However, one has to note that these fluorescent proteins have a half-life of about 24 h (Li et al., 1998), and thus, cannot be ideally used to study temporal dynamics; this could be overcome by using fast degradable fluorescent proteins. On the other hand, the persistence of fluorescent proteins can also be used advantageously for short-term lineage tracing and assessment of cell fate.

Zebrafish mutants with loss of function phenotypes such as the *no isthmus* (*noi*), *acerebellar* (*ace*), and *spiel-ohne-grenzen* (*spg*), were identified from large-scale mutagenesis screens and have been fundamental in elucidating the core gene network that regulates MHB formation and maintenance (Brand et al., 1996; Schier et al., 1996; Lun and Brand, 1998; Reifers et al., 1998; Reim and Brand, 2002). The underlying mutations are strong loss of function or null alleles that result in the survival of these mutants for only a few days after birth. Furthermore, very little is known about the expression patterns of the core MHB genes in the larval and adult brains and even less is known about their function under homeostatic and regenerative conditions. Thus, generating CreER driver lines using this knock-in strategy for important players of several gene families involved in MHB development (Otx, Gbx, Wnt, Fgf8, Pax, Eng) will facilitate loss-/gain-of- function studies that can be spatially and temporally tracked. We show that *otx2:*venus marks some of the neuronal and radial glial cells in the optic tectum and that *pax2a:*venus labels neurons in the valvula cerebelli of the hindbrain and other neural nuclei in the midbrain. However, further studies using various marker combinations are required to completely map the cell types that express Otx2 or Pax2.

## Author contributions

GK and MB conceived the project and designed experiments. GK generated the transgenic zebrafish lines and characterized them in embryonic stages, AC generated data on the adult zebrafish brain, and AM generated reagents for *in situ* hybridization, acquired, and analyzed data from adult brain sections and performed q-RT PCR experiments. GK, AC, and MB wrote the manuscript.

### Conflict of interest statement

The authors declare that the research was conducted in the absence of any commercial or financial relationships that could be construed as a potential conflict of interest.

## Supplementary material

Supplementary Table S1 Table showing indel efficiency and germ-line transmission efficiency for all the transgenic lines.

Supplementary Table S2 List of PCR primers.
