# Supplementary material for: CRISPR/Cas9-Mediated Zebrafish Knock-in as a Novel Strategy to Study Midbrain-Hindbrain Boundary Development
Source: Front Neuroanat. 2017 Jun 30;11:52. doi: 10.3389/fnana.2017.00052 (PMC5492657; doi:10.3389/fnana.2017.00052)
Supplement: Supplementary Table S1 — Table showing indel efficiency and germ-line transmission efficiency for all the transgenic lines. [file Table1.DOCX]

**Supplementary table 1**

| **Transgenic line** | **Indel efficiency for sgRNA** | **Germ-line transmission efficiency (based on successful reporter expression)** |
| --- | --- | --- |
| *otx2*:venus | 6/10= 60% | 2/50= 4% |
| *otx2*:tRFP |  | 2/26= 7.6% |
| *Pax2a*:venus | 8/10= 80% | 2/10= 20% |
| *Pax2a*:tRFP |  | 2/70= 2.8% |
