# Supplementary material for: CRISPR/Cas9-Mediated Zebrafish Knock-in as a Novel Strategy to Study Midbrain-Hindbrain Boundary Development
Source: Front Neuroanat. 2017 Jun 30;11:52. doi: 10.3389/fnana.2017.00052 (PMC5492657; doi:10.3389/fnana.2017.00052)
Supplement: Supplementary Table S2 — List of PCR primers. [file Table2.DOCX]

**Supplementary table 2**

List of PCR primers

Primers

| Gene | Forward | Reverse |
| --- | --- | --- |
| *otx2*-Bait | GGGTGACGCTGAACTTATGTTCACC | TTTACCCCCCACAACCATCTTTAGC |
| *pax2a*-Bait | TAACAAGCCTCGATTTGACAACG | GATAATCGACTGAGGTCGCCG |
| *otx2*:venus 5’junction (A+B) | GACAGATTGGGAGGCGAAAC | ACTTGTGGCCGTTTACGTCG |
| *otx2*:venus 3’junction (C+D) | CCGAAATCGGCAAAATCCCT | CTCACCTGGGTATCCAACGG |
| *otx2*:tRFP 5’junction (A+B) | GACAGATTGGGAGGCGAAAC | GATCTTCATGGTCTGGGTGC |
| *otx2*:tRFP 5’junction (C+D) | TTGCTGGCCTTTTGCTCACA | CTCACCTGGGTATCCAACGG |
| *Pax2a*:venus 5’junction (A+B) | ATCAAAAGCGGCGGTAATGC | CTTGTAGTTGCCGTCGTCCT |
| *Pax2a*:venus 3’junction (C+D) | CCTTTTGCTGGCCTTTTGCT | CGCGCACATGTAAGCCTATG |
| *Pax2a*:tRFP 5’junction (A+B) | CATCCGACATCAATACCTATAAACG | AAGTGGTGGTTGTTCACGGT |
| *Pax2a*:tRFP 5’junction (C+D) | TTGCTGGCCTTTTGCTCACA | CGCGCACATGTAAGCCTATG |
| *otx2 Insitu (adult)* | CCTGGAAATTCCAAGTGTTGTGA | TGAACGGGGACAGACAGTTC |
| *otx2* (Q-RT PCR) | TTAACGGGCATCGGCTTGAA | TCCTTAGCGCACTTCTGCTT |
| *pax2a* (Q-RT PCR) | ACCCCACCTCTACACTTGCT | GGAACTTAATAACGCGGGGT |
| *Beta actin* (Q-RT PCR) | CCTTCCTGGGTATGGAATCT | GACAGCACTGTGTTGGCATA |
